# The Molecular Biology of Wound Healing

**DOI:** 10.1371/journal.pbio.0020278

**Published:** 2004-07-20

Anyone who's endured their share of childhood scrapes has probably heard some version of the motherly admonishment, “Don't pick that scab, you'll just make it worse!” It turns out, Mom was on to something. Tissue damage in humans triggers a well-characterized response marked by rapid blood clotting and a recruitment of epidermal cells to the injury. When you remove a scab, you're also removing some of the newly regenerated tissues growing underneath, thereby interfering with the healing process.

Many different cell types and proteins have been linked to the repair process, but the complexity of the mammalian wound response has challenged efforts to determine their individual roles. Only the first step—the signaling cascade that promotes blood clotting—is understood at the molecular level. Since dissecting the wound response pathway at the molecular level requires an organism that lends itself to relatively easy genetic manipulations, Michael Galko and Mark Krasnow of Stanford University turned to the quintessential genetics organism, *Drosophila melanogaster*, to create a novel system for studying wound healing. After stabbing fruitfly larvae with a needle to create a nonfatal puncture wound, Galko and Krasnow then analyzed significant morphological, cellular, and molecular events at various stages of healing.

Immediately after wounding, the larvae begin to bleed, and about 15 minutes after wounding, the wound darkens and a “plug” of cellular debris forms in the wound. The plug staunches the bleeding and provides a protective layer. Within two hours the plug's outer layer forms a dark, tough scab that presumably serves as an effective barrier to further blood loss. Two or three days later, the healing process is complete.

Galko and Krasnow observed the activity of epidermal cells during healing by labeling their nuclei with a fluorescent protein and staining their membranes. Soon after wounding, cells along the wound margin elongated and aligned themselves toward the wound, then fused together to form a large, multinucleate (multiple nuclei) epidermal cell surrounding the scab. Over the next six hours, these cells also spread along and through the plug, re-establishing a continuous epidermis.

Since this epidermal spreading resembles that seen during a developmental stage of the fruitfly, where the process depends on the JNK signaling pathway, the authors investigated JNK signaling to shed light on the genetics and cellular events of healing. Sure enough, they found that the JNK pathway was activated during the peak hours of wound healing. Inhibiting the pathway in fly mutants—the classic approach in fly genetics—had dramatic effects on the wound-healing process. The early stages of healing—including plug and scab formation—weren't affected, but epidermal spreading to regenerate the intact epidermis was either blocked or defective. In contrast, larvae with defects in a gene required for the generation of crystal cells—a type of blood cell implicated in processes linked to scab formation—could not properly form scabs. In these scabless wounds, the JNK pathway was hyperactive, epidermal cells at the wound's margin started to spread to close the wound but often failed, and the wound did not heal. Score one for Mom.

But apparently Mom wasn't totally right: As Galko and Krasnow discovered, a scab isn't always necessary. When the authors pinched larvae without puncturing the overlying cuticle, the wounds did not form scabs. However, they still saw many of the same processes at work around this “pinch” wound that they saw around the puncture wound, and the pinch wounds healed efficiently. This indicates, the authors argue, that the scab functions primarily to provide stability to the wound site and help restore tissue integrity when both the epidermis and overlying cuticle are damaged during wounding.

While the stages of wound healing outlined here occur at a particular time and place, these results suggest that each stage is controlled by distinct genetic programs and signaling pathways triggered by the wound. Since many aspects of the fly wound response resemble those in mammals, it's likely that the molecular components are also shared. That makes identifying the molecular underpinnings of wound healing a high research priority. And thanks to the powerful system presented here, this task should be all the easier.

